# Effect of the lysosomotropic agent chloroquine on mTORC1 activation and protein synthesis in human skeletal muscle

**DOI:** 10.1186/s12986-021-00585-w

**Published:** 2021-06-12

**Authors:** Michael S. Borack, Jared M. Dickinson, Christopher S. Fry, Paul T. Reidy, Melissa M. Markofski, Rachel R. Deer, Kristofer Jennings, Elena Volpi, Blake B. Rasmussen

**Affiliations:** 1 Division of Rehabilitation Sciences, University of Texas Medical Branch, 301 University Blvd, Galveston, TX 77555-1124 USA; 2 Department of Nutrition and Metabolism, University of Texas Medical Branch, 301 University Blvd, Galveston, TX 77555-1124 USA; 3 Department of Internal Medicine/Geriatrics, University of Texas Medical Branch, 301 University Blvd, Galveston, TX 77555-1124 USA; 4 Sealy Center On Aging, University of Texas Medical Branch, 301 University Blvd, Galveston, TX 77555-1124 USA; 5 Department of Preventive Medicine and Population Health, University of Texas Medical Branch, 301 University Blvd, Galveston, TX 77555-1124 USA; 6 Present Address: Center for the Study of Aging and Human Development, Duke University, Durham, NC USA; 7 Present Address: Department of Health Sciences, Central Washington University, Ellensburg, WA USA; 8 Present Address: Department of Athletic Training and Clinical Nutrition, University of Kentucky, Lexington, KY USA; 9 Present Address: Department of Kinesiology, Nutrition and Health, Miami of Ohio University, Oxford, OH USA; 10 Present Address: Department of Health and Human Performance, University of Houston, Houston, TX USA; 11 Present Address: Department of Biostatistics, University of Texas MD Anderson Cancer Center, Houston, TX USA

**Keywords:** Amino acid sensing, Muscle protein turnover, mTOR signaling, Chloroquine

## Abstract

**Background:**

Previous work in HEK-293 cells demonstrated the importance of amino acid-induced mTORC1 translocation to the lysosomal surface for stimulating mTORC1 kinase activity and protein synthesis. This study tested the conservation of this amino acid sensing mechanism in human skeletal muscle by treating subjects with chloroquine—a lysosomotropic agent that induces in vitro and in vivo lysosome dysfunction.

**Methods:**

mTORC1 signaling and muscle protein synthesis (MPS) were determined in vivo in a randomized controlled trial of 14 subjects (10 M, 4 F; 26 ± 4 year) that ingested 10 g of essential amino acids (EAA) after receiving 750 mg of chloroquine (CHQ, *n* = 7) or serving as controls (CON, *n* = 7; no chloroquine). Additionally, differentiated C2C12 cells were used to assess mTORC1 signaling and myotube protein synthesis (MyPS) in the presence and absence of leucine and the lysosomotropic agent chloroquine.

**Results:**

mTORC1, S6K1, 4E-BP1 and rpS6 phosphorylation increased in both CON and CHQ 1 h post EAA ingestion (*P* < 0.05). MPS increased similarly in both groups (CON, *P* = 0.06; CHQ, *P* < 0.05). In contrast, in C2C12 cells, 1 mM leucine increased mTORC1 and S6K1 phosphorylation (*P* < 0.05), which was inhibited by 2 mg/ml chloroquine. Chloroquine (2 mg/ml) was sufficient to disrupt mTORC1 signaling, and MyPS.

**Conclusions:**

Chloroquine did not inhibit amino acid-induced activation of mTORC1 signaling and skeletal MPS in humans as it does in C2C12 muscle cells. Therefore, different in vivo experimental approaches are required for confirming the precise role of the lysosome and amino acid sensing in human skeletal muscle.

*Trial registration* NCT00891696. Registered 29 April 2009.

## Background

Various anabolic stimuli facilitate muscle growth through the stimulation of the mechanistic target of rapamycin complex 1 (mTORC1). In particular, activation of mTORC1 results in the induction of a signaling cascade that promotes the enhancement of protein initiation and translation [1–3]. While ingestion of protein or amino acids has been shown to result in increases in skeletal muscle protein synthesis in animal and human models [4–8], the precise mechanism(s) through which amino acids activate mTORC1 is less understood.

An increase in amino acid availability within the cell shifts the environment from a catabolic to an anabolic state. During catabolism, mTORC1 is inactive. The inactive state of mTORC1 coincides with an upregulation of autophagy through the lysosomal degradation pathway [9]. This increase in autophagy provides amino acids that may be converted into energy-yielding substrates during periods of low energy availability. Conversely, during the anabolic state, autophagy is suppressed, and the lysosome becomes an integral component in the synthesis of new proteins via mTORC1 activation [10].

Recent work in human embryonic kidney cells (HEK) has identified important molecular processes through which amino acids are “sensed” at the cellular level. During periods of low energy availability, mTORC1 is inactive and the GATOR1 protein complex serves as a GTPase-activating protein (GAP) to inactivate Rag A/B proteins located on the surface of the lysosome. These Rag proteins, in a GDP-bound state, prevent localization of mTORC1 to the lysosome [11, 12]. On the other hand, increased amino acid availability within the cell activates the amino acid sensing machinery upon introduction of the amino acids into the lysosome. The arginine transporter SLC38A9 may be the first amino acid transporter identified that signals availability of amino acids to the lysosome [13, 14]. Once amino acids are “sensed” by the cell, GATOR1 action is inhibited via GATOR2 activation through a mechanism by which Sestrin 2 serves as a leucine sensor [15, 16]. This allows for the Rag proteins to switch from a GDP-bound state to a GTP-bound state. The Rag A/C heterodimer becomes active in the GTP-bound state and then recruits the mTORC1 complex to bind to the lysosome. This colocalization of mTORC1 and the lysosome initiates the mTORC1 signaling cascade [12] resulting in a stimulation of protein synthesis during amino acid sufficiency [17].

The mechanism of amino acid sensing via mTOR/lysosomal colocalization was discovered in HEK cells. Whether a similar mechanism is responsible for activating mTORC1 in the presence of increased amino acid availability within human skeletal muscle is not known. A substantial amount of research has demonstrated the link between protein or amino acid ingestion and increases in skeletal muscle protein synthesis. To this point, these studies have been descriptive in nature. This proposed mechanism could shed light on the long-sought mechanism bridging the gap between amino acid ingestion and mTOR pathway-activated protein synthesis. Currently there is evidence to suggest that colocalization of mTORC1 and the lysosome occurs in skeletal muscle in the presence of amino acids [18, 19] We were intrigued by the drug chloroquine, a lysosomotropic agent that causes lysosomal dysfunction in vivo, as to whether it would be a useful pharmacological intervention to test the role of the lysosome in amino acid sensing in human skeletal muscle. Therefore, we hypothesized that amino acid-induced activation of mTORC1 signaling and muscle protein synthesis would be inhibited by the administration of chloroquine. To test our hypothesis, we employed a randomized controlled human trial followed by an in vitro cell culture study.

## Methods

### Clinical trial

#### Screening of participants

We recruited fourteen healthy, men and women 18–40 years of age for this randomized clinical trial. Participant characteristics are shown in Table 1.

**Table 1:** Subject characteristics

	N	Gender	Age, years	BMI, kg/m^2^	Fat %	Lean Mass, kg
CON	7	5 M, 2F	24.9 ± 1.5	22.0 ± 0.8	21.3 ± 2.1	47.4 ± 2.9
CHQ	7	5 M, 2F	26.9 ± 1.9	23.0 ± 1.0	22.0 ± 2.2	50.3 ± 4.1

Data are mean ± SEM

**Fig. 1:**
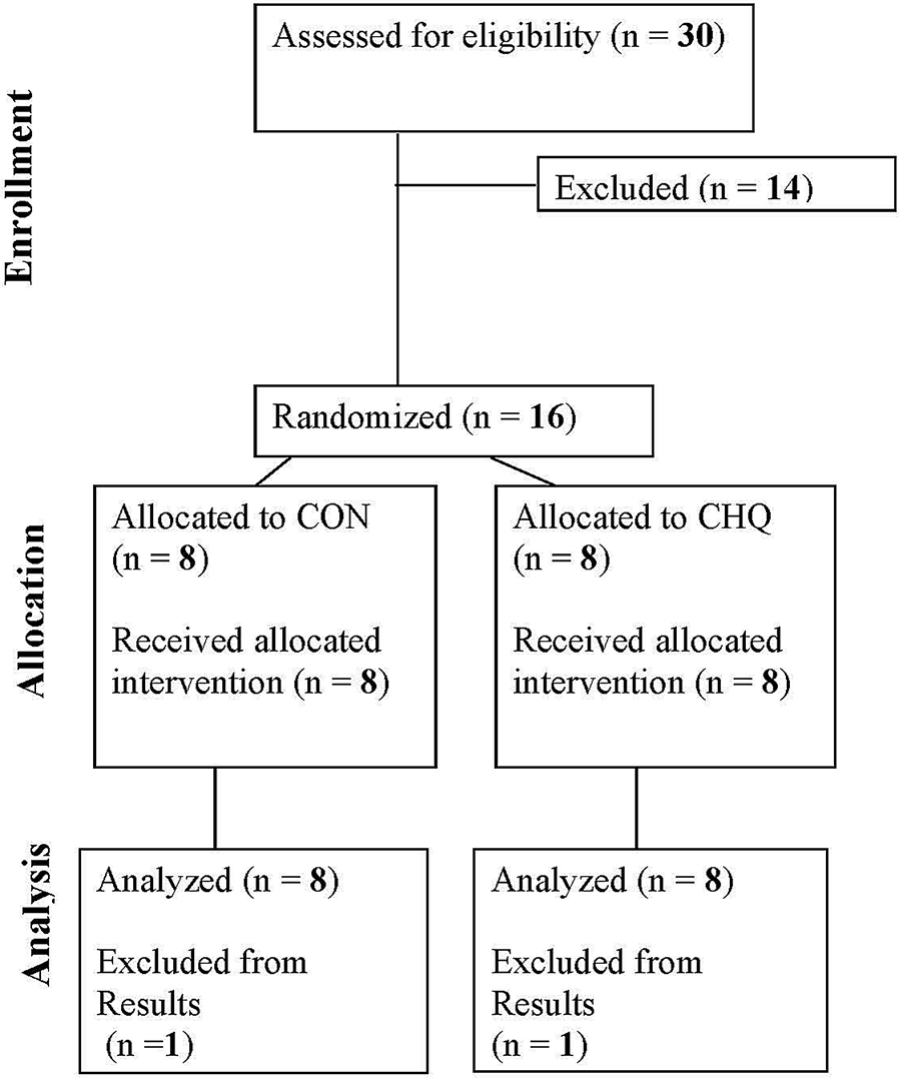
Consort flow diagram for study recruitment

The participants were recruited through flyers, newspaper advertisements, and word of mouth (Fig. 1). Participants were required to be healthy, only recreationally active, nonsmoking, with a body mass index < 30. Participants were screened at the Institute for Translational Sciences-Clinical Research Center (ITS-CRC) at the University of Texas Medical Branch. The screening included: laboratory tests (complete blood count with differential, liver and kidney function tests, coagulation profile, fasting blood glucose, hepatitis B and C screening, HIV test, thyroid stimulating hormone, lipid profile, urinalysis, and drug screening), clinical history with physical exam, and a dual-energy X-ray absorptiometry (DXA) scan (Lunar iDXA, GE Healthcare, Madison, WI) for measuring lean and fat mass. All participants provided written informed consent before enrollment in the study. The study was approved by the Institutional Review Board of the University of Texas Medical Branch, and is in compliance with the Declaration of Helsinki as revised in 1983.

### Study design

**Fig. 2:**
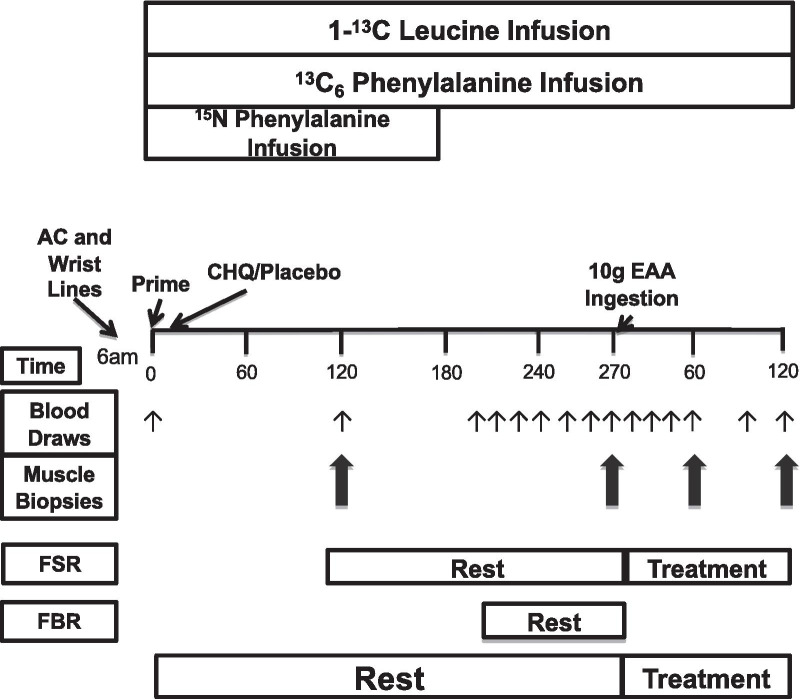
Schematic of randomized experimental protocol. Participants ingested 10 g EAA following biopsy two. The small arrows represent blood draws whereas the large arrows represent biopsies. EAA, essential amino acids. FSR, fractional synthesis rate. FBR, fractional breakdown rate. n = 7 (CON), 7 (CHQ)

In a randomized controlled trial, subjects were randomized to either a control or treatment group, control (N = 7 Con) or chloroquine (N = 7 CHQ). Both groups completed an identical experimental trial (see below) with the exception of the treatment group receiving chloroquine prior to ingesting EAA (Fig. 2). One subject per group was excluded from the results due to an inability to retrieve a muscle sample during the third biopsy. Enrolled participants reported to the ITS-CRC at ~ 1800 h the night before the study. Participants refrained from exercise for at least 48 h prior to admission. Participants were fed a standardized dinner (10 kcal/kg of body weight; 60% carbohydrate, 20% fat, and 20% protein) and a snack at 2100 h (5 kcal/kg of body weight; 60% carbohydrate, 20% fat, and 20% protein), and asked to sleep in the UTMB CRC. After 2300 h, they were allowed only water until the completion of the experimental trial. CHQ subjects ingested a 250 mg dose of chloroquine at 2000 h the night before the study and a 500 mg dose the next morning following commencement of the stable isotope tracers consistent with De feo et al. [20]. Control subjects received nothing. No placebo pill was given as it seemed unnecessary as the subjects remained in bed for the duration of the study and could not manipulate the outcome measures of this study via a placebo effect.

### Experimental protocol

On the morning of the experimental trial, an 18G polyethylene catheter was inserted into a vein in the antecubital space in order to begin the primed, constant infusion (~ 10 h) of L-[ring-^13^C_6_] phenylalanine and L-[^15^N] phenylalanine (Sigma-Aldrich, St. Louis, MO, USA). A background blood sample was taken prior to commencement of the isotope infusion. The priming dose for the labeled phenylalanine was 2 μmol∙kg^−1^ while the infusion rate was 0.05 μmol kg^−1^ min^−1^. A retrograde catheter was inserted into a heated hand vein on the contralateral arm so that arterialized blood may be taken for sampling. Muscle biopsies were performed on the lateral aspect of the *vastus lateralis* for the determination of resting mixed muscle fractional synthesis rate (FSR) at 2 h and 4.5 h following stable isotope infusion initiation. All biopsies were taken with a 5 mm Bergström biopsy needle under sterile procedure and local anesthesia (1% lidocaine). The EAA beverage was consumed following the second biopsy with biopsies three and four performed 60 min and 120 min post ingestion respectively in order to measure post EAA mTORC1 signaling and protein synthesis. A single incision was used for both pre EAA muscle biopsies while a second incision was used for the two post EAA biopsies. Multiple sampling from the same area was limited by separating the incisions by ~ 7 cm. Biopsies taken from the same incision were angled ~ 5 cm from the previous one. This method has been utilized in both our lab [21, 22] and as well as others [23, 24]. Muscle tissue was immediately blotted, frozen in liquid nitrogen and stored at − 80 °C until analysis. Blood samples were collected during the resting (0, 89, 95, 105, 115, 125, 135, 150 min) and post-ingestion (0, 15, 30, 45, 60, 75, 90, 105, 120 min) time periods for the determination of blood L-[ring-^13^C_6_] phenylalanine enrichment and amino acid concentrations. The infusion study concluded with the fourth muscle biopsy at which time the participants were fed a standard meal.

### Essential amino acid beverage

The EAA beverage was consumed following biopsy two. The ingested EAA were dissolved in 300 mL of Fresca® and enriched (8%) with L-[ring-^13^C_6_] and L-[^15^N] phenylalanine to maintain isotopic steady state in arterialized blood. The composition of the beverage is shown in Table 2.

**Table 2:** Composition of the essential amino acid solution

	% of total	Grams (g)
Histidine	11	1.1000
Isoleucine	10	1.0000
Leucine	18	1.8500
Lysine	16	1.5500
Methionine	3	0.3000
Phenylalanine	16	1.5500
Threonine	14	1.4500
Valine	12	1.2000

### Muscle protein turnover

Enrichments of free L-[ring-^13^C_6_]Phenylalanine, and L-[^15^N]Phenylalanine in blood and tissue fluid were measured by gas chromatography–mass spectrometry (GC–MS) after addition of appropriate internal standards and precipitation of blood and tissue proteins with sulfosalycilic acid, extraction with cation exchange chromatography, and tert-butyldimethylsilyl derivatization (t-BDMS). Correction for skewed isotopomer distribution and overlapping spectra were performed as previously described [24]. The incorporation of L-[ring-^13^C_6_]Phenylalanine in the mixed muscle proteins was measured after protein extraction and hydrolysis, amino acid extraction with cation exchange chromatography, TBDMS derivatization, and GC–MS analysis [25, 26].

### Calculation of muscle protein synthesis

Muscle proteins and intracellular free amino acids were extracted from biopsy samples as previously described [21]. Bound muscle and intracellular free concentrations were calculated with the internal standard method using tracer enrichments for L-[ring-^13^C_6_] phenylalanine, L-[^15^N] phenylalanine and appropriate internal standards via GC–MS (6890 Plus CG, 5973N MSD, 7683 autosampler, Agilent Technologies, Palo Alto, CA). Measurements were determined as previously described [24, 26]. Mixed-muscle protein-bound phenylalanine enrichment was analyzed by GCMS after protein hydrolysis and amino acid extraction, [27] using the external standard curve approach [28]. The FSR of mixed muscle proteins was calculated from the incorporation rate of L-[ring-^13^C_6_] phenylalanine into the mixed muscle proteins, and the free-tissue phenylalanine enrichment where ΔE_P_/t is the slope of the straight line that fits the protein-bound phenylalanine enrichment across two sequential biopsies, t is the time interval encompassing the two biopsies, E_M(1)_, and E_M(2)_ are the phenylalanine enrichments (tracer/tracee) in the free muscle pool in the two biopsies. The results are presented as %/h. Phenylalanine is used because it is an essential amino acid that is not oxidized in the muscle tissue. Thus, phenylalanine utilization in the muscle is an index of muscle protein synthesis seen in the following equation:

$${\text{FSR}} = (\Delta {\text{E}}_{{\text{p}}} /t)/[({\text{E}}_{{{\text{M}}(1)}} + {\text{E}}_{{{\text{M}}(2)}} )/2] \cdot 60 \cdot 100$$

### Calculation of muscle protein breakdown

Muscle protein fractional breakdown rate (FBR) was measured with phenylalanine tracers using the precursor-product method [24]. The method requires measurement of intracellular free phenylalanine enrichment at steady-state and after 1 h of tracer decay. Frequent arterialized blood sampling during that 1 h period is necessary for tracking the decay of blood enrichment. To measure FBR at baseline, the L-[ring-^13^C_6_] phenylalanine enrichment at 4 h was used as the plateau enrichment and L-[^15^N] Phenylalanine enrichment at 4 h was used for the 1 h decay enrichment. FBR was calculated using the formula:

$$FBR = {{\Delta E_{M} } \mathord{/ {\vphantom {{\Delta E_{M} } {[p\int {E_{A} (t)dt - (1 + p)\int {E_{M} (t)dt] \cdot (Q_{M} /T)} } }}} \kern-\nulldelimiterspace} {[p\int {E_{A} (t)dt - (1 + p)\int {E_{M} (t)dt] \cdot (Q_{M} /T)} } }}$$

where EA(t) and EM(t) are the arterialized and muscle free enrichments at time t, and t1 and t2 are two time points. *P* = EM/(EA − EM) at plateau, EA and EM are enrichments in the arterial pool and muscle intracellular pool, respectively, and QM/T is the ratio of free to bound phenylalanine in muscle.

### Whole body proteolysis

Whole body proteolysis was measured by dividing the L-[ring-^13^C_6_] phenylalanine tracer infusion rate by the blood L-[ring-^13^C_6_] phenylalanine enrichment (tracer to tracee ratio) at each given time point [29].

### Western blot analysis

Phosphorylation of mTORC1, 4E-BP1, S6K1, and rpS6 was measured using western blot techniques as previously described [22]. 50 μg of protein from each sample was loaded in duplicate onto a 7.5% or 15% polyacrylamide gel (Criterion; Bio-Rad) and subjected to electrophoresis at 150 V for 70 min. Following electrophoresis, proteins were transferred to a polyvinylidene difluoride membrane (Bio-Rad) that were then blocked in 5% non-fat dried milk. Membranes were incubated with a primary antibody overnight at 4 °C. The following rabbit polyclonal primary antibodies (Cell Signaling, Beverley, MA) were used: mTOR (Ser^2448^), S6K1 (Thr^389^), 4EBP1 (Thr^37/46^), and ribosomal protein S6 (Ser^240/244^). Blots were incubated with secondary antibody (Amersham Bioscience) washed, and then a chemiluminescent solution (ECL plus; Amersham BioSciences, Piscataway, NJ, USA) was administered. Optical density measurements were then obtained with a digital imager (Bio-Rad) so that a densitometric analysis (Quantity One software, version 4.5.2; Bio-Rad) could be performed. Following detection of the phosphorylated protein, blots were stripped of primary and secondary antibodies and then re-probed for other proteins. All data is expressed relative to the internal standardized rodent skeletal muscle control used to normalize across blots.

### Cell culture

Murine C2C12 myoblasts were obtained from American Type Culture Collection (Manassas, VA) and cultured on 0.1% gelatin‐(Sigma‐Aldrich, St. Louis, MO) coated 6-well cultureware plates in growth media (high‐glucose Dulbecco's modified Eagle medium supplemented with 10% fetal bovine serum, 50 U of penicillin/mL, 50 μg of streptomycin/mL; Invitrogen, Carlsbad, CA). The cells were incubated in an atmosphere of 5% CO2/95% air at 37 °C. At ~ 90% confluency, differentiation medium (low‐glucose Dulbecco's modified Eagle medium supplemented with 2% horse serum, 50 U of penicillin/mL, 50 μg of streptomycin/mL; Invitrogen, Carlsbad, CA) was added to cultures for approximately 5 days to allow for formation of multinucleated myotubes.

### Cell culture experimental design

#### mTORC1 signaling

At the outset of the experiment, myotubes were nutrient starved for 8 h in starvation media of HEPES‐buffered saline with no leucine (HBS, 20 mmol/L HEPES/Na, 140 mmol/L NaCl, 2.5 mmol/L MgSO4, 5 mmol/L KCl, and 1 mmol/L CaCl2; pH 7.4; Sigma‐Aldrich). For each 6-well plate, two wells were designated “control (Con)” and allowed to be starved for an additional 70 min in starvation media for a total of 9 h and 10 min. Two wells were designated “leucine only (Leu)”. These wells were starved for a total of 9 h and then administered 1 mM leucine for 10 min. Lastly, two wells were designated “chloroquine plus leucine (Chq + leu)”. These wells were nutrient starved for 8 h and then administered 2 mg/ml chloroquine for 60 min followed by 1 mM leucine treatment for 10 min. The experimental procedure was repeated to reach a sample size of eight per group. All wells were washed with PBS between all treatment administrations. This specific protocol was developed as a result of extensive pilot testing in order to properly optimize these experimental conditions. The 2 mg/ml dose was chosen following MTT cell viability assays.

Following treatments, myotubes were rinsed with PBS and each well scraped in ice‐cold extraction buffer (50 mmol/L Tris‐HCl, 250 mmol/L mannitol, 50 mmol/L NaF, 5 mmol/L Na pyrophosphate, 1 mmol/L EDTA, 1 mmol/L EGTA, 1% Triton X‐100, 1 mmol/L DTT, 1 mmol/L benzamidine, 0.1 mmol/L PMSF, 5 μg/mL soybean trypsin inhibitor, pH 7.4). Samples were frozen in liquid nitrogen until analyzed.

To determine protein concentration, samples were thawed and vortexed three times and later sonicated for 15 s. Protein concentrations were calculated using the Bradford Protein Assay (Smartspec Plus, Bio‐Rad, Hercules, CA).

### Myotube protein synthesis

MyPS was measured using the surface sensing of translation (SUnSET) technique as described by Goodman [30]. Cells were designated into two conditions, “nutrient rich” and “nutrient starve”. Within each condition, one subset was administered chloroquine while the other was not and thus served as controls. For the starvation conditions, myotubes were starved and treated as described above for mTORC1 signaling with the exception of no leucine administration. Thus nutrient starve control cells were starved for 9 h in starvation media. Nutrient starve chloroquine cells were starved in starvation media for 8 h, then for an additional hour in starvation media with 2 mg/ml chloroquine. Cells were then administered 1 µM puromycin (Thermo Fisher Scientific, Wilmington, DE) for 30 min and collected as described above. The nutrient rich condition entailed a 16 h serum starve (no horse serum) in low glucose DMEM followed by a 1 h starvation in HEPES‐buffered saline containing 2 mg/ml chloroquine for cells in that subgroup. Cells were then put back into DMEM while provided 1 µM puromycin for 30 min and subsequently collected. The experimental procedure was repeated to reach a sample size of six per group. The experimental procedure here differs from the mTORC1 signaling procedure due to technical limitations. It was not possible to detect a difference between control and a 10 min 1mM leucine administration using this method. We believe that a 1 mM bolus of leucine can result in mTORC1 pathway phosphorylation, yet it is not sufficient to promote activation of protein synthesis at a level necessary for detection. This is most likely due to the limited availability of amino acids required for generating new proteins as these cells are starved for 9 h with no amino acids and then given only leucine. Therefore a serum starve in low glucose DMEM was used to provide those amino acids in the nutrient rich groups.

### Western blot analysis

Cell lysates were diluted (1:1) in a 2 × sample buffer mixture (125 mmol/L Tris, pH 6.8, 25% glycerol, 2.5% SDS, 2.5% β‐mercaptoethanol, and 0.002% bromophenol blue) and then boiled for 3 min at 100 °C. Equal amounts of total protein were loaded into each lane, and the samples were separated by electrophoresis at 150 V for 60 min on a 7.5% polyacrylamide gel (Criterion, Bio‐Rad). All samples were loaded in duplicate with a loading control and molecular weight ladder (Precision Plus, Bio‐Rad).

Following electrophoresis, the protein was transferred to a polyvinylidene difluoride membrane (Bio‐rad) at 50 V for 60 min. Blots were blocked in 1% bovine serum albumin for 1 h for mTORC 1 signaling proteins or 30 min for puromycin and then incubated with primary antibody overnight at 4 °C. The following rabbit polyclonal primary antibodies for mTORC1 signaling (Cell Signaling, Beverley, MA) were used, mTOR (Ser2448) and S6K1 (Thr389). The primary antibody for puromycin protein synthesis was the mouse IgG2a monoclonal anti-puromycin antibody clone 12D10 (EMD Millipore, Billerica, MA).The following morning, secondary antibody (Amersham Bioscience Piscataway, NJ for mTOR signaling), or horseradish peroxidase conjugated anti-mouse IgG Fc 2a antibody (Jackson ImmunoResearch Laboratories Inc., West Grove, PA, USA for puromycin protein synthesis) was added for 1 h at room temperature. Blots were incubated in a chemiluminescent solution (ECL plus, Amersham BioSciences,) for 5 min and optical density measurements quantified using a digital imager (ChemiDoc, Bio‐Rad) and densitometric analysis was performed using Quantity One 4.5.2 software (Bio‐Rad). Membranes were stripped using Restore Western Blot Stripping buffer (Pierce Biotechnology, Rockford, IL). Phosphorylation values were normalized to the loading control.

### Statistical analysis

All values are expressed as Mean ± SEM. Cell culture experimental data were modeled with t-tests to test differences between groups. Clinical trial data were transformed using the Box-Cox set of transformations to stabilize the variance and make the data approximately normally distributed. To test differences between groups, the data were modeled using an ANCOVA model with resting/baseline values as a covariate. The testing of differences was thus accomplished through a t-test of the parameter indicating the difference between groups. Comparisons with resting values were based on testing contrasts across time using a mixed model with subject as a random intercept term. All baseline comparisons were done using two-group t-tests. Fold changes were tested against baseline using a one-sample t-test. Significance was set at *P* < 0.05. All calculations were done in R version 13.2 [31].

## Results

### Clinical trial

#### Subject characteristics

Descriptive characteristics for all subjects are shown in Table 1. The participants in both groups displayed similar lean mass, percent body fat and BMI (*P* > 0.05). The consort diagram for clinical trial enrollment is shown in Fig. 1.

### Blood and muscle amino acid concentrations

**Fig. 3:**
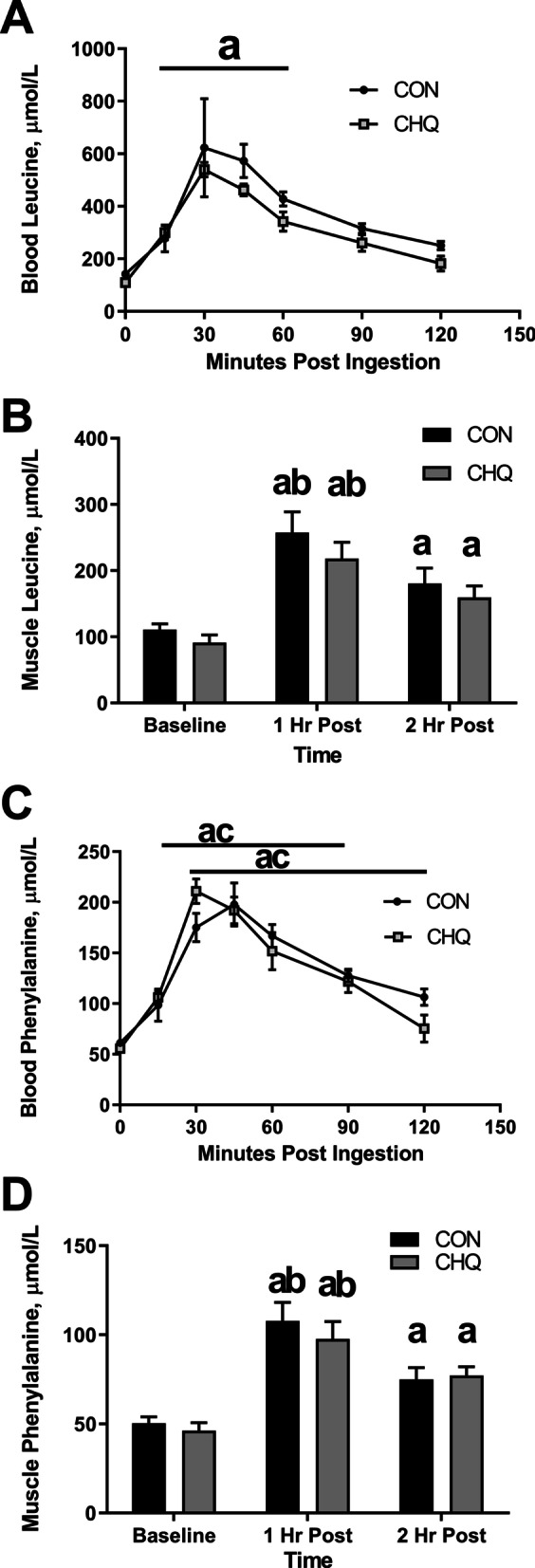
Blood and Muscle Amino Acid Concentrations. Changes from rest in blood Leucine (**A**), muscle Leucine (**B**), blood Phenylalanine (**C**), and muscle Phenylalanine (**D**) at baseline and following ingestion of a 10 g EAA beverage. Data are mean ± SEM. N = 7 for both treatment groups. “a” different from resting values, *P* < 0.05. “b” difference between 1 h post and 2 h post, “c” difference between treatment groups, *P* < 0.05. CON, control. CHQ, chloroquine

Blood concentrations for leucine (Fig. 3A) were elevated from baseline (*P* < 0.05) for both treatment groups 60 min post ingestion. Leucine intracellular muscle concentrations were elevated in both groups at 1 h and at 2 h post ingestion compared to baseline (*P* < 0.05) while 1 h post ingestion was significantly different (*P* < 0.05) than 2 h post ingestion for both groups (Fig. 3B). There was no difference between groups for either leucine measure (*P* > 0.05). Phenylalanine concentrations in the blood were elevated from baseline (*P* < 0.05) for CON from 30 to 120 min post ingestion and CHQ from 15 to 90 min post ingestion (*P* < 0.05) (Fig. 3C). Phenylalanine intracellular muscle concentrations were elevated in both groups at 1 h and at 2 h post ingestion compared to baseline (*P* < 0.05) while 1 h post ingestion was significantly higher than 2 h post ingestion for both groups (Fig. 3D). There was no difference between groups for either phenylalanine measure.

### Muscle mTORC1 signaling

**Fig. 4:**
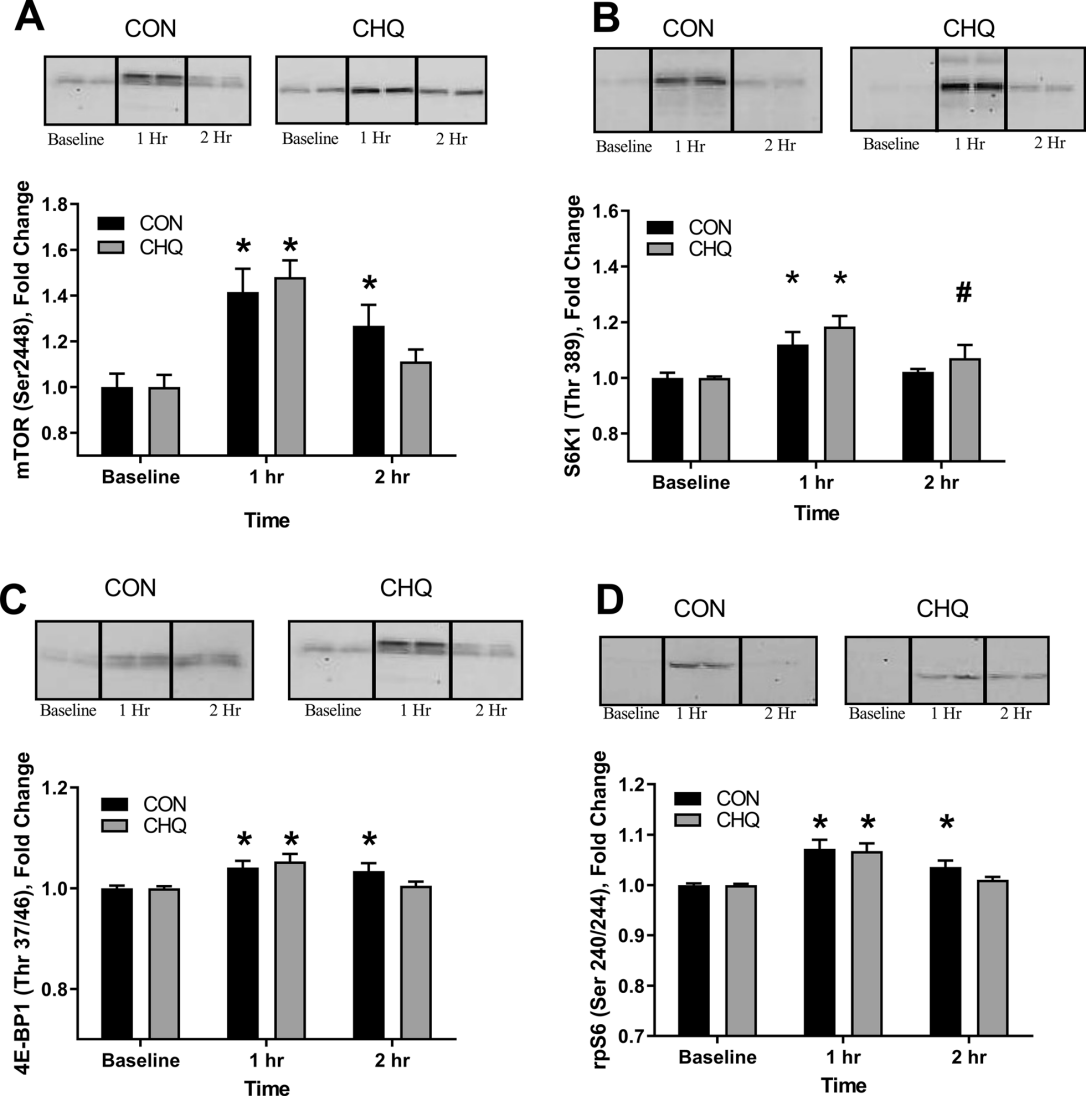
Muscle mTORC1 Signaling. Western blot analyses of mTORC1 (**A**) and mTOR pathway-related proteins: S6K1 (**B**), 4EBP1 (**C**) and rpS6 (**D**), at baseline and following a 10 g EAA beverage. Data are mean ± SEM. N = 7 for both treatment groups. *Different from pre, *P* < 0.05. #trend difference from pre, *P* = 0.07. CON, control. CHQ, chloroquine

The phosphorylation status of mTORC1 (Ser2448) was significantly increased (*P* < 0.05) at 1 h post ingestion in both groups compared to baseline with only CON significantly elevated at 2 h compared to baseline (Fig. 4A). S6K1 (Thr389) phosphorylation was elevated at 1 h post ingestion for both groups. At 2 h post ingestion, CHQ showed a trend for increased S6K1 phosphorylation of *P* = 0.07 compared to baseline (Fig. 4B). 4E-BP1 (Thr37/46) phosphorylation was significantly increased (*P* < 0.05) at 1 h post ingestion in both groups compared to baseline. CON 4E-BP1 phosphorylation was significantly elevated at 2 h compared to baseline (Fig. 4C). The phosphorylation status of rpS6 (Ser240/244) was significantly increased (*P* < 0.05) at 1 h post ingestion in both groups compared to baseline. Only CON rpS6 phosphorylation was significantly elevated at 2 h compared to baseline (Fig. 4D). There were no differences in total protein abundance between groups at any time point for all measured proteins (*P* > 0.05).

### Fractional synthetic rate

**Fig. 5:**
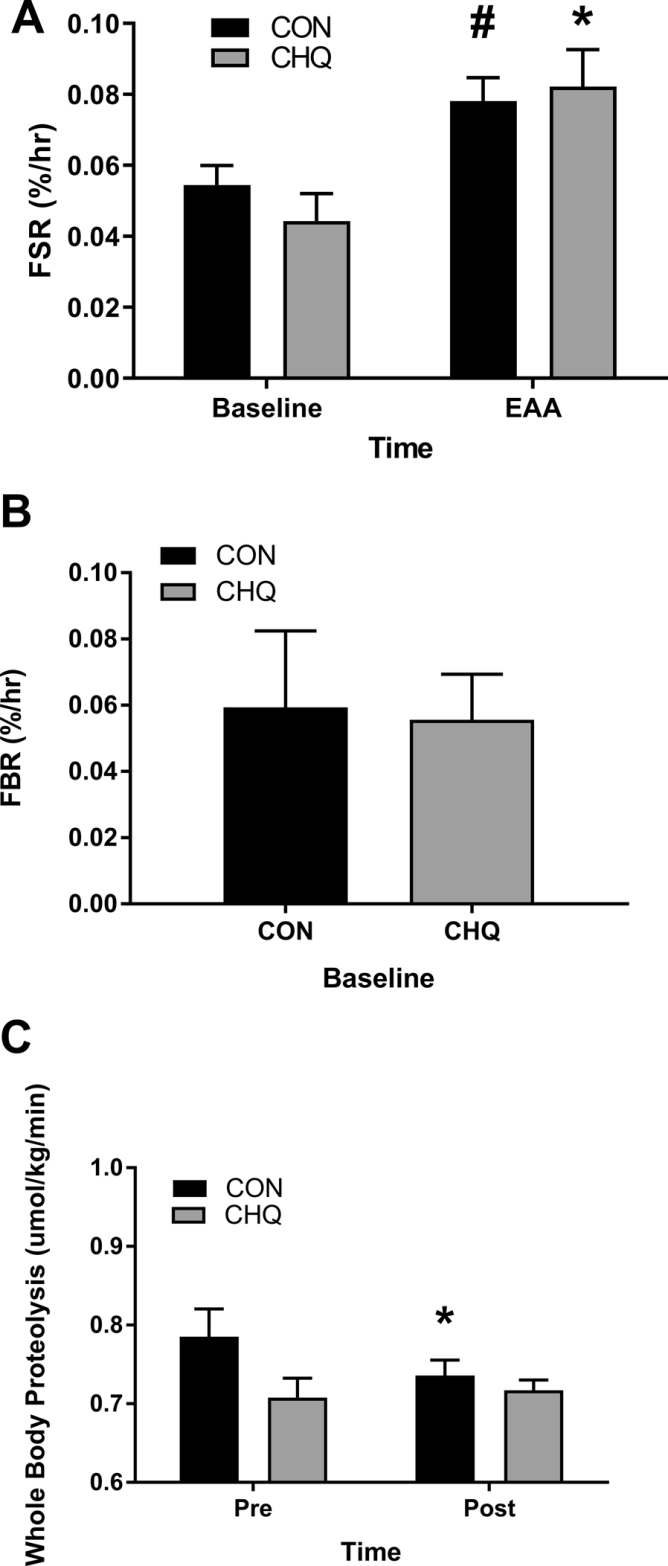
Fractional Synthetic Rate, Fractional Breakdown Rate, and Whole Body Proteolysis. **A** FSR (vastus lateralis) at baseline and for the two hour period post ingestion of the 10 g EAA beverage. Data are mean ± SEM. N = 7 for both treatment groups. *Different from rest, *P* < 0.05. #trend difference from pre, *P* = 0.06. FSR, fractional synthesis rate. CON, control. CHQ, chloroquine. EAA, essential amino acids. **B** FBR (vastus lateralis) for the 1 h period prior to ingestion of the 10 g EAA beverage. Data are mean ± SEM. N = 7 for both treatment groups. FBR, fractional breakdown rate. CON, control. CHQ, chloroquine. **C** Whole body proteolysis at baseline and for the two hour period post ingestion of the 10 g EAA beverage. Data are mean ± SEM. N = 7 for both treatment groups. *Different from pre, *P* < 0.05. CON, control. CHQ, chloroquine

There was no difference between groups for muscle protein synthesis at any time point (*P* > 0.05). Post EAA ingestion FSR was elevated from resting values for CHQ (*P* < 0.05) while CON showed a trend for an increase *P* = 0.06 (Fig. 5).

### Fractional breakdown rate

There was no difference (*P* > 0.05) between groups for muscle protein breakdown at rest (Fig. 5).

### Whole body proteolysis

There was no difference between groups at any time point (*P* > 0.05). Whole body proteolysis was significantly decreased from baseline in CON following EAA ingestion (*P* < 0.05) (Fig. 5).

### Cell culture experiments

#### Myotube mTORC1 signaling and protein synthesis

**Fig. 6:**
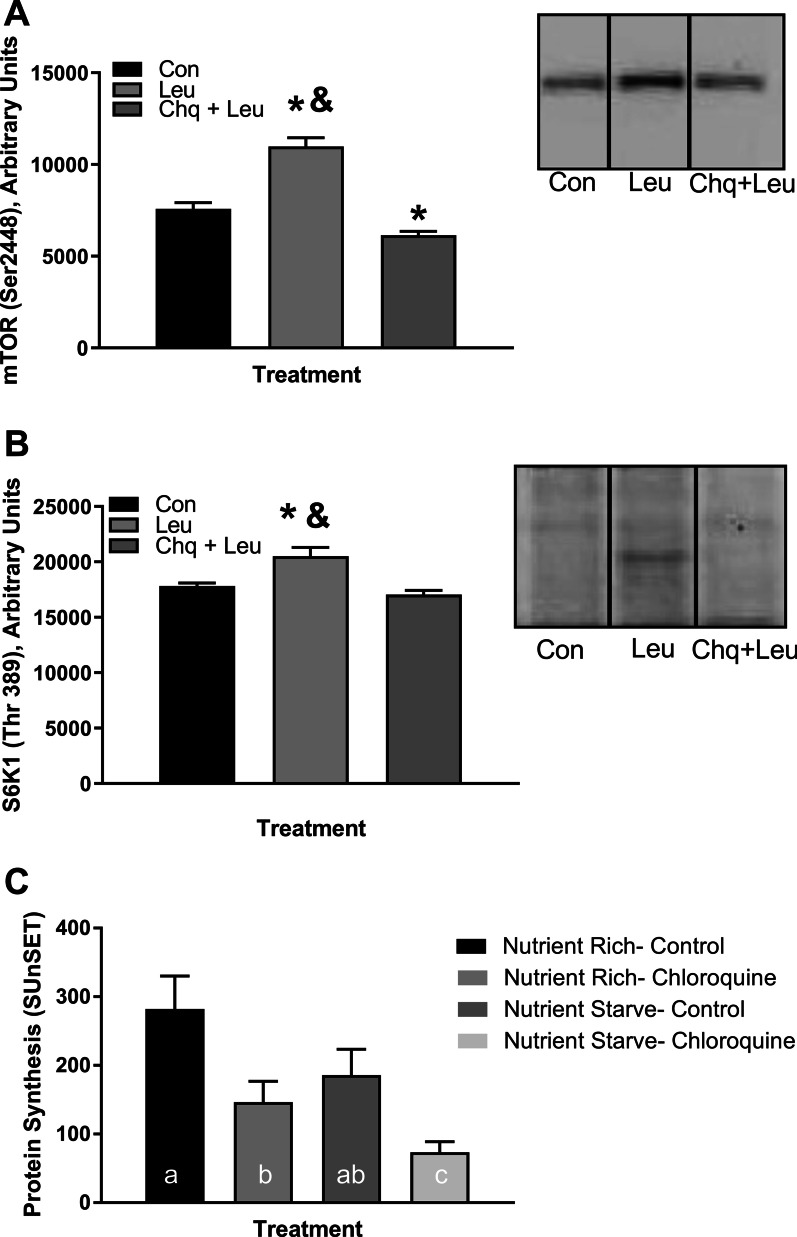
Myotube mTORC1 signaling and protein synthesis. Cells were starved for 8 h in HEPES-buffered saline. Control was starved for an additional 70 min in fresh HEPES. “Leucine” was starved for 60 min in fresh Hepes and 10 min in fresh Hepes with leucine. “Chloroquine + leucine” was starved in fresh HEPES with chloroquine for 60 min and 10 min in fresh HEPES with leucine. **A** Phosphorylation of mTORC1 at Ser 2448 in control, leucine (1 mM for 10 min), and chloroquine + leucine (2 mg/ml chloroquine for 60 min followed by 1 mM leucine for 10 min) conditions. Insert shows representative western blot for each condition. **B** Phosphorylation of S6K1 at Thr 389 (same conditions). Insert shows representative western blot for each condition. Data are mean ± SEM. N = 8 for both treatment groups. *Different from control, *P* < 0.05. &Different from chloroquine + leucine. Chq, chloroquine. **C** Western blot analyses of protein synthesis using the SUnSET technique. Nutrient Rich-Control and Chloroquine were serum starved for 16 h and nutrient starved for 1 h (2 mg/ml for Chloroquine group only) prior to 30 min puromycin (1 µM) exposure. Nutrient Starve groups were starved under the same conditions as described above for mTORC1 signaling prior to 30 min puromycin (1 µM) exposure. Data are mean ± SEM, N = 6. ^abc^Columns with uncommon letters differ, *P* < 0.05

The phosphorylation status of mTORC1 (Ser 2448) was significantly increased with 1 mM leucine administration (*P* < 0.05) compared to control and chloroquine + leucine (Fig. 6A). The phosphorylation status of S6K1 (Thr389) was significantly increased with 1 mM leucine administration (*P* < 0.05) compared to control and chloroquine + leucine (Fig. 6B). MyPS was reduced in the presence of chloroquine for both the nutrient rich and nutrient starved conditions (*P* < 0.05) compared to their respective controls. There was no difference in protein synthesis between the two control conditions (nutrient rich vs. nutrient starve) (*P* = 0.095) nor was there a difference between control in the nutrient starvation state and chloroquine in the nutrient rich state (*P* = 0.478). Nutrient rich control was different compared to nutrient starved chloroquine (*P* < 0.05) (Fig. 6C).

## Discussion

We conducted an in vivo human clinical trial wherein we administered chloroquine prior to ingesting essential amino acids to test the effect of chloroquine on mTORC1 signaling and protein synthesis. We found no effect of chloroquine on either outcome measure following EAA ingestion during the clinical trial.

The nutritional benefits of amino acid supplementation in humans have been investigated for many decades going at least as far back as the 1940s [32]. Since that time, it has become understood that amino acids promote protein synthesis [33]. Yet the mechanism(s) underlying amino acid activation of protein synthesis have been elusive until recently. Work in HEK cells has demonstrated the necessity of the interaction of the lysosome with the mTOR complex via a complex named Ragulator. The Ragulator protein complex resides on the surface of the lysosome. In the presence of amino acids, Ragulator recruits the Rag proteins in their GTP-bound state to the lysosomal membrane in order to dock with the mTOR complex and initiate mTOR signaling [34, 35]. A year later, [10, 36] revealed an additional component of amino acid sensing, the vacuolar H( +)-adenosine triphosphatase ATPase (v-ATPase). The v-ATPase was shown to provide a critical interaction with the scaffolding protein Ragulator during amino acid activation of mTOR signaling [36]. Other players in the amino acid sensing mechanism have since been identified. GATOR1 is an inhibitor of this pathway as it acts on the GTP-bound Rag proteins. GATOR2 works to inhibit GATOR1 in the presence of amino acids. Lastly the Sestrins are a family of proteins that interact with GATOR2 and are necessary for the colocalization of the lysosome and mTOR [37].

As all of the research described above was conducted in kidney cells, this current study sought to test the conservation of this amino acid sensing mechanism in human skeletal muscle. Settembre et al. [38] demonstrated reduced mTOR signaling with the inhibition of downstream target S6K1 using the drug chloroquine [38]. Chloroquine is a lysosomotropic agent that raises the internal pH of the lysosome. This change in pH causes lysosomal dysfunction and inhibition of lysosomal protein degradation [39]. Yu and Long [40] also demonstrated lysosomal/autophagy dysfunction through chloroquine administration in C212 cells [40]. This inhibition resulted in a reduction in mTOR signaling under starvation conditions and an increase in signaling with amino acid supplementation, in the presence of chloroquine, although the treatment included thirteen separate amino acids [40].

To test the role of the lysosome in amino acid sensing in human muscle, we conducted a human trial utilizing a 10 g EAA solution that has been shown to enhance mTORC1 signaling and muscle protein synthesis during previous studies [17, 41]. We found that chloroquine administration did not alter amino acid concentrations in the blood following EAA ingestion and did not inhibit mTORC1 activation. Similarly chloroquine administration did not prevent the EAA-induced increase in muscle protein synthesis in our human subjects. Therefore, we could not confirm the validity of amino acid sensing through the lysosome in humans from this study. We also examined muscle protein breakdown and proteolysis to determine whether any chloroquine-mediated effects were present. Previous work has shown that a 750 mg dose of chloroquine (the dose used in the current study) is sufficient to reduce whole body proteolysis in humans [20]. In our study, we also found that the rate of whole body proteolysis was numerically less in the chloroquine group (e.g., 0.71 v 0.79 μmol/kg/min) at baseline, but this difference did not reach statistical significance. Amino acids have been shown to reduce both whole body as well as muscle protein breakdown [42, 43], and our data showed a similar response in the control group. Interestingly, whole body proteolysis did not drop in response to EAA ingestion for the chloroquine group as it did for the control group which may indicate that chloroquine was having an impact on whole body proteolysis. Nevertheless, muscle protein breakdown was not altered by chloroquine in our samples. Similarly Barrett et al. did not witness a change in skeletal muscle protein breakdown during a 3 h chloroquine infusion into the forearm of human subjects [44]. Therefore, we are able to surmise two potential explanations for these results. The first is that inhibition of the lysosome by chloroquine can inhibit mTORC1 signaling and FSR in skeletal muscle as hypothesized, but the dosage of chloroquine used in our study was not sufficient to alter muscle protein turnover. Chloroquine is known to accumulate in certain tissues of the body, specifically the liver, spleen, kidney and lung. These organs were found to have chloroquine concentrations 200–500 times that found in the blood [45]. Therefore, it is possible that the dosage for this study was insufficient to reach the levels necessary to interrupt lysosomal function within skeletal muscle. That would help to explain the disparity between our results at the whole body level versus at the muscle specific level as well as differences between earlier studies performed in various cell lines, and our study performed in humans. A second possibility is that lysosomal disruption within human skeletal muscle does not alter mTORC1 signaling and muscle protein turnover following amino acid ingestion in vivo. It is possible that mTORC1 signaling may remain intact following lysosomal disruption by an amino acid sensing mechanism that is independent of the lysosome in humans. We acknowledge the chloroquine dosing as a limitation, however, administering larger doses was not an option as that would have been considered unsafe to our participants (as determined by our study physician and local institutional review board).

Following completion of the clinical trial, we conducted in vitro experiments to test the effect of chloroquine on mTORC1 signaling and protein synthesis in C2C12 myotubes. We found an increase in both mTORC1 and S6K1 phosphorylation following ten minutes of 1 mM leucine administration. This increase was not seen in cells provided 2 mg of chloroquine for 1 h prior to leucine administration. Therefore it would appear that mTORC1 signaling is potentially hampered in muscle cells in the presence of chloroquine. While it is not possible to determine if lysosomal disruption is the sole cause of this diminished signaling, it does provide evidence that is comparable to that seen by others mentioned above [38, 40]. Protein synthesis was reduced by chloroquine regardless of nutrient condition. Therefore, we can tentatively conclude from these data that this mechanism of amino acid sensing (i.e., mTORC1 translocation to the lysosomal membrane in response to amino acid availability) is conserved in C2C12 skeletal muscle cells.

## Conclusions

In conclusion, chloroquine did not inhibit amino acid-induced activation of mTORC1 signaling and skeletal muscle protein synthesis in humans as it does in C2C12 muscle cells. Therefore, different in vivo experimental approaches are required for confirming the precise role of the lysosome and amino acid sensing in human skeletal muscle.

## Data Availability

The datasets used and/or analyzed during the current study available from the corresponding author on reasonable request.

## Abbreviations

BMI: Body mass index
CHQ: Chloroquine
Chq + leu: Chloroquine plus leucine
CON: Control
DXA: Dual-energy X-ray absorptiometry
EAA: Essential amino acids
FSR: Fractional synthesis rate
FBR: Muscle protein fractional breakdown rate
GAP: GTPase-activating protein
GC–MS: Gas chromatography–mass spectrometry
HEK: Human embryonic kidney
ITS-CRC: Institute for Translational Sciences-Clinical Research Center
Leu: Leucine only
MPS: Muscle protein synthesis
mTORC1: Mechanistic target of rapamycin complex 1
MyPS: Myotube protein synthesis
SEM: Standard error of the mean
SUnSET: Surface sensing of translation
